# Association between hearing loss and cognitive decline in the elderly: A systematic review with meta-analysis study

**DOI:** 10.1371/journal.pone.0288099

**Published:** 2023-11-09

**Authors:** Débora Conceição Santos de Oliveira, Isaac Suzart Gomes-Filho, Edna Maria Araújo, Michelle de Santana Xavier Ramos, Julita Maria Freitas Coelho, Adan Araújo Marques, Alexandre Marcelo Hintz, Dóris Firmino Rabelo, Ana Claudia Morais Godoy Figueiredo, Simone Seixas da Cruz

**Affiliations:** 1 Health Sciences Center, Federal University of Recôncavo of Bahia, Bahia, Brazil; 2 Department of Health, Feira de Santana State University, Bahia, Brazil; 3 Federal Institute of Education of Bahia—Lauro de Freitas, Bahia, Brazil; 4 Epidemiology Surveillance, Federal District Health State Secretariat, Brasilia, Distrito Federal, Brazil; Federal University of Paraiba, BRAZIL

## Abstract

**Objective:**

Hearing loss has been pointed out as a potential predictor for cognitive decline. This study conducted a systematic review to evaluate the scientific evidence on the association between hearing loss in the elderly and cognitive decline, as well as whether race/color influences this relationship.

**Method:**

The search for studies was performed in the following electronic databases: MedLine/PubMed Web of Science, Scopus and Virtual Health Library, and MedRkiv up to August 2022. Studies with epidemiological designs that assess the association between hearing loss and cognitive decline in the elderly were eligible for inclusion. Three independent reviewers performed the selection, data extraction and evaluation of the quality of the studies using the Newcastle-Ottawa Scale. A meta-analysis using a random effects model estimated the global association measurements (Beta coefficient: β) and their 95% confidence intervals (95%CI), and the Higgins and Thompson indicator (I^2^) was also estimated to assess statistical heterogeneity among the studies.

**Results:**

5,207 records were identified in the database surveys, of which only 18 were eligible studies, totaling 19,551 individuals. Hearing loss was associated with cognitive decline in the elderly, with statistical significance: β = -0.13; 95%CI = -0.23 to -0.04; I^2^ = 98.70%). For black individuals, the magnitude of the association increased: β = -0.64; 95%CI = -3.36 to 2.07; I^2^ = 95.65%, but it was not statistically significant.

**Conclusion:**

The findings of this systematic review showed the existence of a significant relationship between hearing loss and cognitive decline in the elderly, as well as signaling that among black individuals the magnitude of the association can be increased.

## Introduction

Projections indicate that, by 2050, life expectancy will be 82 years for men and 86 for women in developing countries [1]. In Brazil, studies by the Brazilian Institute of Geography and Statistics projected that by 2060, a quarter of the population will be over 65 years old [2]. This fact engenders complex challenges in taking care of the health of the older person, including their hearing capacity, due to the inversion of the age pyramid.

Hearing loss among the elderly reduces quality of life, due to a decrease in daily activities and sociability, and may generate processes of loss of autonomy, social isolation, depression, and dementia resulting from a cognitive decline that seems to accompany it [3–6].

Impaired hearing and cognition are disabling conditions for the elderly. The impairment of cognitive functions is directly related to an individual´s reduced interaction in their social context, hindering daily life due to detrimental changes to the domains of attention, perception, executive functions, capacity for retention and application of knowledge in daily life [7]. So far, the effect of using hearing aids on cognitive functions is still unclear, since reports on the effects of using hearing aids have produced inconclusive results, but it is known that hearing aid use can affect immediate cognitive function [8] and there is recent evidence that long-term use may delay cognitive decline [9].

Cognitive decline has been indicated as a predictor of severe cognitive diseases such as dementia and can affect up to 33% of older adults aged 85 years and over [10,11]. This scenario is further aggravated by racial disparities in health that, often, black older people have faced throughout their lives [12–15]. Furthermore, evidence indicates that cognitive decline is among the most prevalent mental health problems in elderly black individuals [15].

The relationship between hearing loss and cognitive decline has already been investigated by some researchers [16–18]. However, the two problems have a complex chain of causality, so the underlying mechanisms that lead to the connection between the two are not yet well understood [19,20]. In addition, there is an insufficient number of studies that investigated the association between hearing loss and cognitive decline in minority populations, such as the elderly black population. Racial/ethnic and socioeconomic disparities exist in hearing health care and represent critical areas for research and intervention [21].

For the best of our knowledge, there is no systematic review with meta-analysis with a sample composed exclusively of the elderly. Previous systematic reviews involved mixed populations in relation to age group [17,22–24]. Thereby, the present systematic review with meta-analysis evaluated the scientific evidence on the association between hearing loss and cognitive decline in the elderly, as well as its magnitude among elderly black individuals.

## Method

### Registration and protocol

The protocol of this systematic review was registered in the International Prospective Registry of Systematic Reviews—PROSPERO DATABASE: CRD42022340230. This review followed the PRISMA—2020 Statement [25].

### Eligibility criteria for studies

Cohort, cross-sectional, and case-control studies involving people aged 60 years or older were included. Initially, there was no linguistic restriction, and the data collection was conducted from March 4 up to August 1, 2022. Studies without a clear description of the diagnostic criteria for hearing loss and/or cognitive decline, such as those with self-reported information, were excluded. Studies involving samples of elderly people diagnosed with dementia were also excluded.

### Information sources

The studies were searched using the following electronic databases; Medline/PubMed, Virtual Health Library—VHL, Web of Science and Scopus. The reference lists of the articles selected for systematic review, as well as specific databases containing texts from the gray literature, such as MedRkiv, were also examined.

### Search strategies

The descriptors used and their synonyms were identified in the Medical Subject Headings (Medical Terms Titles)—MeSH. The keywords used for the search strategies were elderly, hearing loss, cognitive dysfunction and cognitive decline. The English terms employed were: Aged, Deafness, Hearing Loss, Cognitive Impairment, Cognitive Dysfunction and Cognitive Decline. The following Boolean operators were employed: AND and OR. The initial search strategy was adapted to the other electronic databases (Chart 1 in S1 File). To assess the quality of search strategies, the Peer Review Electronic Search Strategy—PRESS was used [26].

### Study selection

All phases of this review were carried out independently by 3 reviewers. Disagreements in the evaluated phases were resolved among them [25]. After excluding duplicates, the studies were selected by reading titles and abstracts (D.C.S.O., S.S.C., and A.A.M), using the Rayyan program [27]. The reviewers were unaware of the decisions made by their peers during the selection process of the articles. A.G., A.A.M., and S.S.C. read the full text of the selected articles independently, and those which met the eligibility criteria were included in the systematic review.

### Data extraction

The researchers (D.C.S.O., S.S.C., and A.A.M.) performed data extraction from the included articles using the following fields: author’s name, year of publication, place and year of study, objective, study design, sample size, criteria for the diagnosis of hearing loss and cognitive decline, association measurement, presence of confounding and modifier variables and the main findings.

### Quality of studies

To evaluate the quality of the selected studies, the Newcastle—Ottawa quality assessment scale for cohort, case-control and adapted for cross-sectional studies were used [28,29]. The researchers (D.C.S.O, S.S.C. and A.A.M) performed the quality assessment of all studies, independently, and then the information was confronted until a consensus was reached among them.

### Data analysis

Quantitative data analysis used Stata version17® statistical package (StataCorp LLC, College Station, TX, USA). Higgins and Thompson’s I-square indicator (I^2^) was used to evaluate statistical heterogeneity among studies [30]. To interpret the magnitude of the inconsistency of the data among the studies included in the meta-analysis, the percentage score of test I^2^ was used as follows: 0% to 40%: it may not be important, as it may indicate slight heterogeneity; 30% to 60%: moderate heterogeneity; 50% to 90%: substantial heterogeneity; 75% to 100%: very substantial heterogeneity. The diagnosis of the origin of heterogeneity was also performed in the studies, using visual inspection of the Galbraith chart [31]. Eventual publication biases were evaluated by inspection performed using the of the Begg funnel chart [32].

The selection of statistical methods considered the data of the association measurements between hearing loss and cognitive decline–expressed in the Beta coefficient (*β*) and its 95% confident interval (95%CI) of the linear regression model between the continuous variables of exposure and outcome. The estimates of coefficient β were standardized using Cohen’s d function [33].

## Results

**Fig 1:**
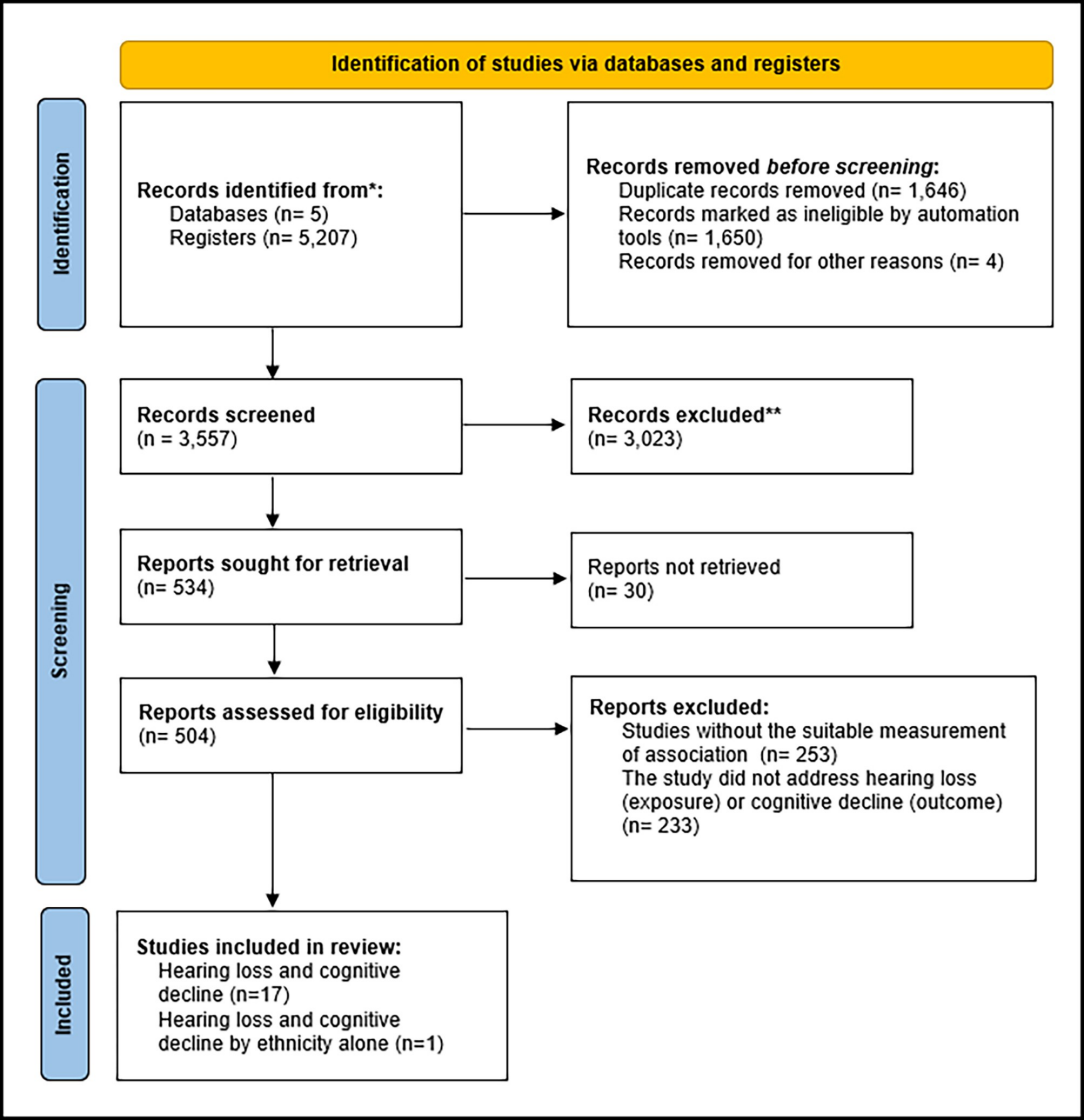
Flowchart of search, selection and inclusión of studies related to the association between hearing loss and cognitive decline in the elderly.

At the end of the search, 5,207 records were identified. Duplicate records were removed for reading titles and summaries. Of these, 504 articles were selected for complete reading and 18 articles met the eligibility criteria of this systematic review (Fig 1).

### Qualitative analysis

**Fig 2:**
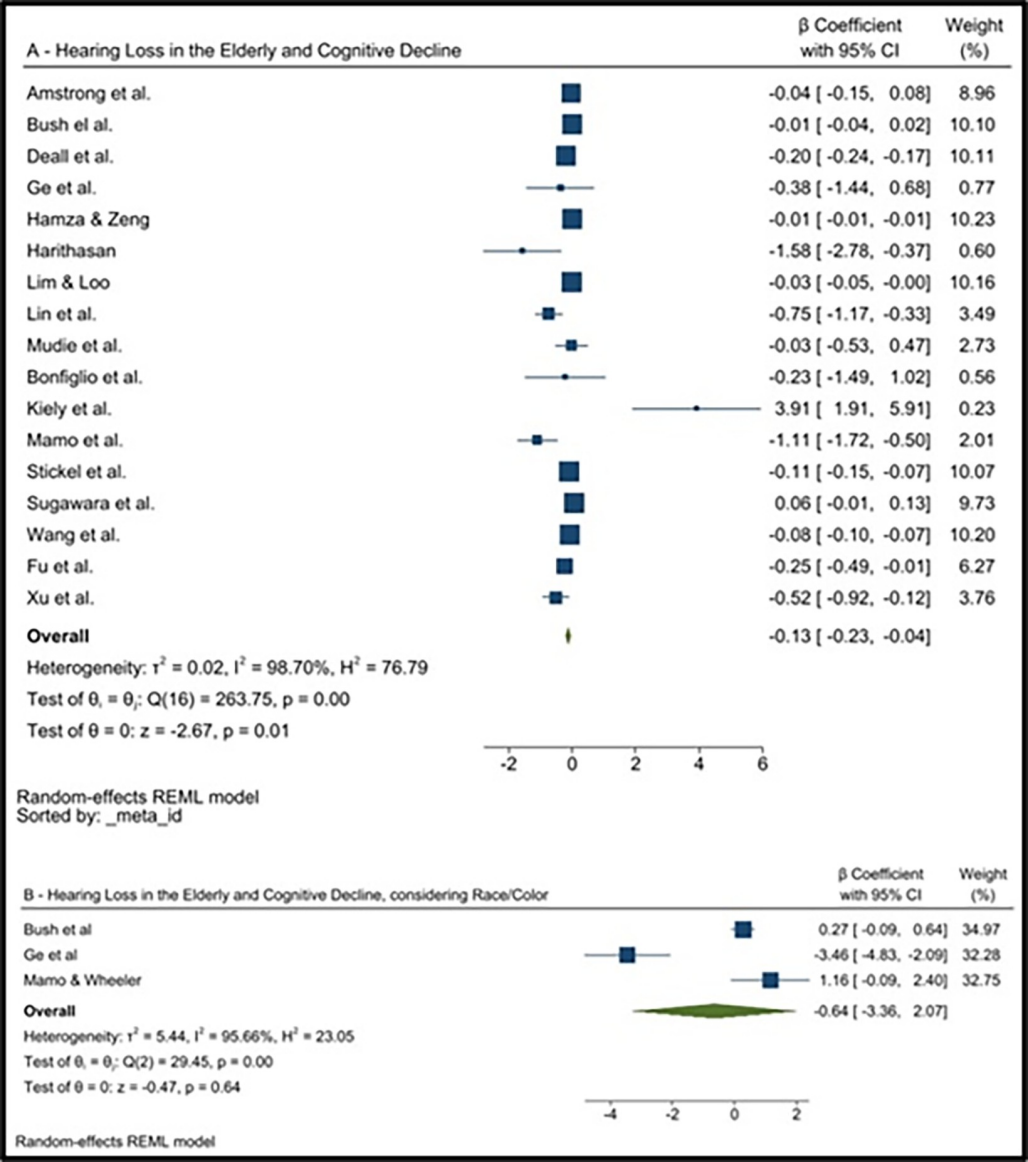
Forest plot of the meta-analysis with association measurements_:_ Beta coefficient (p) and 95% confidence intervals (95%CI)_:_ Between hearing loss and cognitive decline in the elderly, regardless of race/color (N = 17), and among black individuals (N = 3).

The 18 studies considered for this review included 19,551 participants. Of which, for the analysis of the association between hearing loss in the elderly and cognitive decline, regardless of race/color, 17 articles totaled 19,407 elderly people, consisting of meta-analytical model I [34–50] (Fig 2A).

When considering the influence of race/color on the association, only 3 studies were included [37,38,40], with a total of 1,333 participants, representing meta-analytical model II (Fig 2B).

In both models, the investigations were observational. In meta-analytical model I, the number of studies were 6 (35.3%) cross-sectional, 10 (58.8%) cohort and 1 (5.9%) case-control. Most investigations occurred in the North American region 9 (52.9%). The others were developed in Asia.

In addition, in the aforementioned model, 16 (88.2%) presented ≤ 25 dB as a criterion of normal hearing level, 1 (5.9%) ≤ 40 dB and 1 (5.9%) ≤ 20 dB. Regarding the diagnosis of cognitive decline, 10 records (58.2%) used the Mini-Mental State Examination [51]; and 7 (41.2%) used other tests for definition (Table 1).

**Table 1:** General characteristics of the studies used in the meta-analysis without the race/color/ethnicity approach. (N = 17).

Characteristic	N	%
**Study design**		
Cohort	6	35.3
Cross-sectional	10	58.8
Case-control	1	5.9
**Geographic region**		
North America	9	52.9
Asia	8	47.1
**Diagnosis of hearing loss**		
Normal hearing level ≤ 25 dB	15	88.2
Normal hearing level ≤ 40 dB	1	5.9
Normal hearing level ≤ 20 dB	1	5.9
**Diagnosis of cognitive decline**		
Mini-Mental State Examination	10	58.8
Other tests *	7	41.2
**Sample size** **		
≤ 313	9	52.94
> 313	8	47.06
**Methodological quality**		
Moderate: 5–6 points	1	5.9
High: 7–9 points	16	91.4
**Year of publication**		
≤ 2020	10	58.8
> 2020	7	41.2
**Funding**		
Yes	12	70.6
No	2	11.8
Not informed	3	17.6

* Other tests used to diagnose cognitive decline can be found in Chart 2 in S1 File.

** Median as cut-off point.

However, for meta-analytical model II, all studies were conducted in North America 3 (100.0%) and were cross-sectional (Table 1). In this model, the criterion adopted for defining hearing loss was greater than 25 dB in the three studies included. Three different cognitive decline definition criteria were used: Mini-Mental State Examination [52]; a Telephone Interview for Cognitive (TIC) Status [37] and Consortium *to Establish a Registry for Alzheimer’s Disease–*CERAD [38]. Chart 2 in S1 File provides more information about these criteria used to assess cognitive function.

In both models, in general, the evaluation of the quality of the studies was classified as high, with averages of 7.94 (± 0.86). There is variation from moderate to high quality (6 to 9 points), with no article classified as low quality (Charts 3 and 4 in S1 File). In the selected investigations, covariables age and gender were considered as potential confounding factors in all individual studies.

### Hearing loss and cognitive decline

The summary measurement of the meta-analytical model I estimated a significant association between hearing loss and cognitive decline, regardless of race/color (β = -0.13; 95%CI = -0.23 to -0.04), with I^2^ of 98.70% representing high heterogeneity among the studies (Fig 2A). A Galbraith graph was performed, showing some outlier studies but the removal of these did not change either the association measurement or the value of statistical heterogeneity (Fig 1).

The global association measurement of meta-analytical model II showed that among blacks, although the overall measurement was higher than the previous one, statistical significance was not maintained, and heterogeneity remained high: *β* = -0.64; 95%CI = -3.36 to 2.07; I^2^ = 95.66% (Fig 2B).

For the meta-analytical model I, publication bias was investigated and asymmetry in the visual analyses of the included studies were observed (Fig 2).

## Discussion

The main findings of this systematic review with meta-analysis showed that there was an association between hearing loss and cognitive decline in the elderly, regardless of race/color, statistically significant. As far as we know, this is the only systematic review with meta-analysis with a sample composed exclusively of the elderly. The present study presented results similar to those published in previous systematic reviews, which involved mixed populations in relation to age group [17,22–24].

The mechanisms that can promote hearing loss, and, subsequently, become a risk factor for cognitive decline in the elderly, have not yet been completely clarified [17]. Some scholars argue that the degradation of the vascular system in the course of life is an underlying factor, which predisposes the elderly to both hearing loss and cognitive decline, since in the course of life, in general, there is physiological reduction of auditory and brain functions [52].

However, others argue that the reduction of auditory acuity generates difficulties in speech perception, reducing the speed of processing and understanding of language and, consequently, leading the individual to have memory loss, lack of attention and difficulty in developing logical thoughts. According to the above-mentioned theory, there is an important causal relationship between hearing loss and cognitive capacity restriction [53–56].

Furthermore, according to this investigation, in relation to the specific analytical measurement target for black elderly, the findings indicated that the magnitude of the global association measurement (β = -0.64) between hearing loss and cognitive decline was approximately 5 times higher than that estimated in the model in which the item race/color was not considered (β = -0.13). However, there was no statistical significance of the association among black elderly, probably because there were only 3 individual investigations [37,38,40], which presented different characteristics of the population groups involved, with a sample size, which, even having been increased through this meta-analysis, did not have enough power to answer the hypothesis raised.

It is important to highlight that of the synthesis studies that evaluated the association, none of them presented the specific meta-analytical measurement for race/color. The absence of these measurements in previous studies is the main finding of this meta-analysis. Although it is recognized that there are weaknesses in this inference, due to the low statistical power conferred by the insufficient number of studies and the high estimated heterogeneity among them [57].

However, in synthesis investigations, the absence of scientific evidence of quality, according to specific characteristics such as race/color, may be reflections of scarce and limited individual studies on the topic. In other words, the gap in knowledge cannot categorically mean the absence of the influence of race/color on the association between hearing loss and cognitive decline.

This gap may represent an important obstacle to the diagnosis of the auditory health situation of black elderly, since rational decision-making in health, which aims to combat racial inequities, should be based on qualified scientific evidence [58–61].

Although the information resulting from the research, disaggregated by race/color, has been neglected throughout the history of Health Sciences [62], this scenario is changing slowly, in Brazil and in the world. It has already been recognized that the research of health problems by race represents an important step towards the creation of an indispensable process of deconstruction of the centuries old social structure which weakens the black population and promotes the maintenance of health inequities [59,60,62].

Regarding the high heterogeneity identified in the two meta-analysis models, this can be attributed to the different classification systems of cognitive decline, different types of methodological designs and the peculiarities of the sociodemographic characteristics of the population groups that comprised the samples of the individual studies. Most of the selected studies used the Mini-Mental State Examination to diagnose cognitive decline and approximately 40% used other tests that must have contributed greatly to the difference between them. It is known that substantial measurements of heterogeneity in meta-analysis may represent the existence of bias in global estimates and, for this reason, constitute a source of concern with the evidence found [57].

On the other hand, it is noteworthy that the methodological quality of the included studies was generally considered moderate to high, which represents one of the strengths of this meta-analysis [25]. There was an effort on the part of the studies to present adjusted measurements for potential classic confounding, such as age, gender and vascular factors, through the use of multiple analysis, giving greater robustness to the overall results [63].

As positive elements of this systematic review, we mention the insertion of five bibliographic bases, with studies of moderate to high methodological quality—in addition to the use of tools and procedures already validated in the scientific environment for studies of this nature. In this sense, the Peer Review of Electronic Research Strategies (PRESS [26] was used as an instrument to evaluate the quality of research strategies, in an attempt to increase the reliability of the selection of studies.

It was not the objective of this study, but it is suggested that future systematic reviews assess the possible factors that may influence cognitive decline, such as the type of hearing loss, mixed and sensorineural, as well as its laterality, that is, bilateral or predominantly unilateral. These factors are believed to have an impact on cognitive impairment. The accurate classification of hearing loss provides valuable insights into underlying mechanisms, potential risk factors, and appropriate interventions. This will contribute to a better understanding of the diverse effects of hearing loss within specific subgroups of the elderly population and aid in the development of personalized strategies for prevention and intervention. In addition, another relevant issue that needs to be investigated is the effect of using hearing aids on cognitive functions, since it is known that hearing aid use can affect immediate cognitive function [8] and there is recent evidence that long-term use may delay cognitive decline [9,64–66].

Finally, it is important to encourage robust scientific investigations that expand the knowledge about the hypothesis of an association between hearing loss and injuries such as cognitive decline in the elderly, without neglecting the valuable information disaggregated by race/ color, particularly in regions with intense racial inequities. The detailed investigation of the true auditory and general health condition of individuals from different groups is a powerful tool in addressing racial disparities.

## Supporting information

S1 Checklist PRISMA 2020 checklist. (DOCX)

S1 File (DOCX)
