# Supplementary material for: Association between hearing loss and cognitive decline in the elderly: A systematic review with meta-analysis study
Source: PLoS One. 2023 Nov 9;18(11):e0288099. doi: 10.1371/journal.pone.0288099 (PMC10635537; doi:10.1371/journal.pone.0288099)

**SUPPLEMENTARY MATERIAL**

**Chart 1 -** Search strategies according to different electronic databases

| **Medline/PubeMed (04/03/2022)** |
| --- |
| **N=409** |
| (((Aged[Title/Abstract]) OR (Elderly[Title/Abstract])) AND (((((((((((((((((Deafness[Title/Abstract]) OR (Hearing Loss, Complete[Title/Abstract])) OR (Complete Hearing Loss[Title/Abstract])) OR (Hearing Loss, Extreme[Title/Abstract])) OR (Extreme Hearing Loss[Title/Abstract])) OR (Prelingual Deafness[Title/Abstract])) OR (Deafness, Prelingual[Title/Abstract])) OR (Deafness, Acquired[Title/Abstract])) OR (Acquired Deafness[Title/Abstract])) OR (Deafness Permanent[Title/Abstract])) OR (Permanent, Deafness[Title/Abstract])) OR (Permanents, Deafness[Title/Abstract])) OR (Hearing Loss Permanent[Title/Abstract])) OR (Permanent, Hearing Loss[Title/Abstract])) OR (Deaf Mutism[Title/Abstract])) OR (Deaf-Mutism[Title/Abstract])) OR (((((((((((((Hearing Loss[Title/Abstract]) OR (Loss, Hearing[Title/Abstract])) OR (Hypoacusis[Title/Abstract])) OR (Hypoacuses[Title/Abstract])) OR (Hearing Impairment[Title/Abstract])) OR (Deafness, Transitory[Title/Abstract])) OR (Deafnesses, Transitory[Title/Abstract])) OR (Transitory Deafness[Title/Abstract])) OR (Transitory Deafnesses[Title/Abstract])) OR (Transitory Hearing Loss[Title/Abstract])) OR (Hearing Loss, Transitory[Title/Abstract])) OR (Loss, Transitory Hearing[Title/Abstract])) OR (Transitory Hearing Losses[Title/Abstract])))) AND ((((((((((((((((((((((((((((((((((((((((((((((((((((((((((((((((((((((((((((((((((Cognitive Dysfunction[Title/Abstract]) OR (Cognitive Dysfunctions[Title/Abstract])) OR (Dysfunction, Cognitive[Title/Abstract])) OR (Dysfunctions, Cognitive[Title/Abstract])) OR (Cognitive Impairments[Title/Abstract])) OR (Cognitive Impairment[Title/Abstract])) OR (Impairment, Cognitive[Title/Abstract])) OR (Impairments, Cognitive[Title/Abstract])) OR (Mild Cognitive Impairment[Title/Abstract])) OR (Cognitive Impairment, Mild[Title/Abstract])) OR (Cognitive Impairments, Mild[Title/Abstract])) OR (Impairment, Mild Cognitive[Title/Abstract])) OR (Impairments, Mild Cognitive[Title/Abstract])) OR (Mild Cognitive Impairments[Title/Abstract])) OR (Mild Neurocognitive Disorder[Title/Abstract])) OR (Disorder, Mild Neurocognitive[Title/Abstract])) OR (Disorders, Mild Neurocognitive[Title/Abstract])) OR (Mild Neurocognitive Disorders[Title/Abstract])) OR (Neurocognitive Disorder, Mild[Title/Abstract])) OR (Neurocognitive Disorders, Mild[Title/Abstract])) OR (Cognitive Decline[Title/Abstract])) OR (Cognitive Declines[Title/Abstract])) OR (Decline, Cognitive[Title/Abstract])) OR (Declines, Cognitive[Title/Abstract])) OR (Communication Disorders[Title/Abstract])) OR (Communication Disorder[Title/Abstract])) OR (Communicative Disorders[Title/Abstract])) OR (Communicative Disorder[Title/Abstract])) OR (Communication Disorders, Developmental[Title/Abstract])) OR (Communication Disorder, Developmental[Title/Abstract])) OR (Developmental Communication Disorder[Title/Abstract])) OR (Developmental Communication Disorders[Title/Abstract])) OR (Childhood Communication Disorders[Title/Abstract])) OR (Childhood Communication Disorder[Title/Abstract])) OR (Communication Disorder, Childhood[Title/Abstract])) OR (Communication Disorders, Childhood[Title/Abstract])) OR (Communicative Dysfunction[Title/Abstract])) OR (Communicative Dysfunctions[Title/Abstract])) OR (Dysfunction, Communicative[Title/Abstract])) OR (Dysfunctions, Communicative[Title/Abstract])) OR (Neurogenic Communication Disorders[Title/Abstract])) OR (Communication Disorder, Neurogenic[Title/Abstract])) OR (Neurogenic Communication Disorder[Title/Abstract])) OR (Communication Disorders, Neurogenic[Title/Abstract])) OR (Communication Disabilities[Title/Abstract])) OR (Communication Disability[Title/Abstract])) OR (Disabilities, Communication[Title/Abstract])) OR (Disability, Communication[Title/Abstract])) OR (Acquired Communication Disorders[Title/Abstract])) OR (Acquired Communication Disorder[Title/Abstract])) OR (Communication Disorder, Acquired[Title/Abstract])) OR (Communication Disorders, Acquired[Title/Abstract])) OR (Auditory Perceptual Disorders[Title/Abstract])) OR (Auditory Perceptual Disorder[Title/Abstract])) OR (Perceptual Disorder, Auditory[Title/Abstract])) OR (Auditory Processing Disorder[Title/Abstract])) OR (Auditory Processing Disorders[Title/Abstract])) OR (Disorder, Auditory Processing[Title/Abstract])) OR (Disorders, Auditory Processing[Title/Abstract])) OR (Processing Disorder, Auditory[Title/Abstract])) OR (Processing Disorders, Auditory[Title/Abstract])) OR (Psychoacoustical Disorders[Title/Abstract])) OR (Disorder, Psychoacoustical[Title/Abstract])) OR (Disorders, Psychoacoustical[Title/Abstract])) OR (Psychoacoustical Disorder[Title/Abstract])) OR (Acoustic Perceptual Disorder[Title/Abstract])) OR (Acoustic Perceptual Disorders[Title/Abstract])) OR (Disorder, Acoustic Perceptual[Title/Abstract])) OR (Disorders, Acoustic Perceptual[Title/Abstract])) OR (Perceptual Disorder, Acoustic[Title/Abstract])) OR (Perceptual Disorders, Acoustic[Title/Abstract])) OR (Perceptual Disorders, Auditory[Title/Abstract])) OR (Auditory Comprehension Disorder[Title/Abstract])) OR (Auditory Comprehension Disorders[Title/Abstract])) OR (Comprehension Disorder, Auditory[Title/Abstract])) OR (Comprehension Disorders, Auditory[Title/Abstract])) OR (Disorder, Auditory Comprehension[Title/Abstract])) OR (Disorders, Auditory Comprehension[Title/Abstract])) OR (Auditory Inattention[Title/Abstract])) OR (Auditory Inattentions[Title/Abstract])) OR (Inattention, Auditory[Title/Abstract])) OR (Inattentions, Auditory[Title/Abstract])) |
| **Web Of Science (04.03.2022)** |
| **N= 1610** |
| ((#1) AND #2) AND #3  #1  (AB=(Aged )) OR AB=(Elderly)  #2  ((((((((((((((((((((((((((((AB=(Deafness )) OR AB=(Hearing Loss, Complete)) OR AB=(Complete Hearing Loss)) OR AB=(Hearing Loss, Extreme)) OR AB=(Extreme Hearing Loss)) OR AB=(Prelingual Deafness)) OR AB=(Deafness, Prelingual)) OR AB=(Deafness, Acquired)) OR AB=(Acquired Deafness)) OR AB=(Deafness Permanent)) OR AB=(Permanent, Deafness)) OR AB=(Permanents, Deafness)) OR AB=(Hearing Loss Permanent)) OR AB=(Permanent, Hearing Loss)) OR AB=(Deaf Mutism)) OR AB=(Deaf-Mutism)) OR AB=(Hearing Loss )) OR AB=(Loss, Hearing)) OR AB=(Hypoacusis)) OR AB=(Hypoacuses)) OR AB=(Hearing Impairment)) OR AB=(Deafness, Transitory)) OR AB=(Deafnesses, Transitory)) OR AB=(Transitory Deafness)) OR AB=(Transitory Deafnesses)) OR AB=(Transitory Hearing Loss)) OR AB=(Hearing Loss, Transitory)) OR AB=(Loss, Transitory Hearing)) OR AB=(Transitory Hearing Losses)  #3  (((((((((((((((((((((((((((((((((((((((((((((((((((((((((((((((((((((((((((((((((AB=(Cognitive Dysfunction )) OR AB=(Cognitive Dysfunctions)) OR AB=(Dysfunction, Cognitive)) OR AB=(Dysfunctions, Cognitive)) OR AB=(Cognitive Impairments)) OR AB=(Cognitive Impairment)) OR AB=(Impairment, Cognitive)) OR AB=(Impairments, Cognitive)) OR AB=(Mild Cognitive Impairment)) OR AB=(Cognitive Impairment, Mild)) OR AB=(Cognitive Impairments, Mild)) OR AB=(Impairment, Mild Cognitive)) OR AB=(Impairments, Mild Cognitive)) OR AB=(Mild Cognitive Impairments)) OR AB=(Mild Neurocognitive Disorder)) OR AB=(Disorder, Mild Neurocognitive)) OR AB=(Disorders, Mild Neurocognitive)) OR AB=(Mild Neurocognitive Disorders)) OR AB=(Neurocognitive Disorder, Mild)) OR AB=(Neurocognitive Disorders, Mild)) OR AB=(Cognitive Decline)) OR AB=(Cognitive Declines)) OR AB=(Decline, Cognitive)) OR AB=(Declines, Cognitive)) OR AB=(Communication Disorders )) OR AB=(Communication Disorder)) OR AB=(Communicative Disorders)) OR AB=(Communicative Disorder)) OR AB=(Communication Disorders, Developmental)) OR AB=(Communication Disorder, Developmental)) OR AB=(Developmental Communication Disorder)) OR AB=(Developmental Communication Disorders)) OR AB=(Childhood Communication Disorders)) OR AB=(Childhood Communication Disorder)) OR AB=(Communication Disorder, Childhood)) OR AB=(Communication Disorders, Childhood)) OR AB=(Communicative Dysfunction)) OR AB=(Communicative Dysfunctions)) OR AB=(Dysfunction, Communicative)) OR AB=(Dysfunctions, Communicative)) OR AB=(Neurogenic Communication Disorders)) OR AB=(Communication Disorder, Neurogenic)) OR AB=(Neurogenic Communication Disorder)) OR AB=(Communication Disorders, Neurogenic)) OR AB=(Communication Disabilities)) OR AB=(Communication Disability)) OR AB=(Disabilities, Communication)) OR AB=(Disability, Communication)) OR AB=(Acquired Communication Disorders)) OR AB=(Acquired Communication Disorder)) OR AB=(Communication Disorder, Acquired)) OR AB=(Communication Disorders, Acquired)) OR AB=(Auditory Perceptual Disorders )) OR AB=(Auditory Perceptual Disorder)) OR AB=(Perceptual Disorder, Auditory)) OR AB=(Auditory Processing Disorder)) OR AB=(Auditory Processing Disorders)) OR AB=(Disorder, Auditory Processing)) OR AB=(Disorders, Auditory Processing)) OR AB=(Processing Disorder, Auditory)) OR AB=(Processing Disorders, Auditory)) OR AB=(Psychoacoustical Disorders)) OR AB=(Disorder, Psychoacoustical)) OR AB=(Disorders, Psychoacoustical)) OR AB=(Psychoacoustical Disorder)) OR AB=(Acoustic Perceptual Disorder)) OR AB=(Acoustic Perceptual Disorders)) OR AB=(Disorder, Acoustic Perceptual)) OR AB=(Disorders, Acoustic Perceptual)) OR AB=(Perceptual Disorder, Acoustic)) OR AB=(Perceptual Disorders, Acoustic)) OR AB=(Perceptual Disorders, Auditory)) OR AB=(Auditory Comprehension Disorder)) OR AB=(Auditory Comprehension Disorders)) OR AB=(Comprehension Disorder, Auditory)) OR AB=(Comprehension Disorders, Auditory)) OR AB=(Disorder, Auditory Comprehension)) OR AB=(Disorders, Auditory Comprehension)) OR AB=(Auditory Inattention)) OR AB=(Auditory Inattentions)) OR AB=(Inattention, Auditory)) OR AB=(Inattentions, Auditory) |
| **Scopus (04.03.2022)** |
| **N= 3,948** |
| ( ( ( TÍTULO-ABS-KEY ( cognitivo E disfunção ) OU TÍTULO-ABS-KEY ( cognitivo E disfunções ) OU TÍTULO-ABS-KEY ( disfunção E cognitivo ) OU TÍTULO-ABS-KEY ( disfunções E cognitivo ) OU TÍTULO -ABS-KEY ( deficiências cognitivas E ) OU TITLE-ABS-KEY ) OU TITLE-ABS-KEY ( deficiências e ( cognitive AND impairment ) OR TITLE-ABS-KEY ( impairment, AND cognitive ) OR TITLE-ABS-KEY ( impairments, AND cognitive ) OR TITLE-ABS-KEY ( mild AND cognitive AND impairment ) OR TITLE-ABS-KEY ( cognitive AND impairment, AND mild ) OR TITLE-ABS-KEY ( cognitive AND impairments, AND mild leve E cognitivo ) OU TITLE-ABS-KEY ( deficiências E leve E cognitiva ) OU TITLE-ABS-KEY ( leve E cognitiva E deficiências ) OU TITLE-ABS-KEY ( leve E neurocognitivo E transtorno ) OU TITLE-ABS-KEY ( transtorno E leve E neurocognitivo ) OU TÍTULO-ABS-KEY ( transtornos E leve E neurocognitivo ) OU TITLE-ABS-KEY ( leve E neurocognitivo E transtornos ) OU TITLE-ABS-KEY ( neurocognitivo E transtorno E leve ) OU TITLE-ABS-KEY ( neurocognitivo E transtornos E leve ) OU TITLE-ABS- KEY ( cognitivo E declínio ) OU TÍTULO-ABS-KEY ( cognitivo E declínio ) OR TITLE-ABS-KEY ( declínio E cognitivo ) OU TITLE-ABS-KEY ( declínio e cognitivo ) ) OU ( ( TITLE-ABS-KEY ( comunicação E transtornos ) OU TITLE-ABS- KEY ( comunicação E transtorno ) OU TÍTULO-ABS-KEY ( transtornos E comunicativos ) OU TÍTULO-ABS-KEY ( distúrbios E comunicativos ) OU TITLE-ABS-KEY ( comunicação E transtornos, E desenvolvimento ) OU TÍTULO-ABS-KEY ( comunicação E transtorno, E desenvolvimento ) OU TÍTULO-ABS-KEY ( desenvolvimento E comunicação E transtorno ) OU TÍTULO-ABS-KEY ( desenvolvimento E comunicação E transtornos ) OU TÍTULO-ABS-KEY ( infância E comunicação E transtornos ) OU TÍTULO-ABS-KEY ( infância E comunicação E transtorno ) OU TÍTULO-ABS-KEY ( comunicação E transtorno, E infância ) OU TÍTULO-ABS-KEY ( comunicação E transtornos, E infância ) OU TÍTULO-ABS-KEY ( comunicativo E disfunção ) OU TITLE-ABS-KEY ( comunicativo AND ) OU TITLE-ABS-KEYdysfunctions ) OR TITLE-ABS-KEY ( dysfunction, AND communicative ) OR TITLE-ABS-KEY ( dysfunctions, AND communicative ) OR TITLE-ABS-KEY ( neurogenic AND communication AND disorders ) OR TITLE-ABS-KEY ( communication AND disorder, AND neurogenic ) OR TITLE-ABS-KEY ( neurogenic AND communication AND disorder ( comunicação E distúrbios e neurogênicos ) OU TITLE-ABS-KEY ( comunicação E deficiências ) OU TITLE-ABS-KEY ( comunicação E deficiência ) OU TITLE-ABS-KEY ( deficiências E comunicação ) OU TITLE-ABS-KEY ( deficiência , E comunicação ) OU TÍTULO-ABS-KEY ( adquirido E comunicação E distúrbios ) OU TÍTULO-ABS-KEY ( adquirido E comunicação E distúrbio ) OU TÍTULO-ABS-KEY ( comunicação E distúrbio, E adquirido ) OU TÍTULO-ABS-KEY ( comunicação E distúrbios E adquirido ) ) ) OU ( ( TÍTULO-ABS -KEY ( distúrbios E auditivos e perceptivos ) OU TÍTULO -ABS-KEY ( auditivo E perceptivo E transtorno ) OU TÍTULO-ABS-KEY ( perceptivo E transtorno E auditivo ) OU TÍTULO-ABS-KEY ( auditivo E processamento E transtorno ) OU TÍTULO-ABS-KEY ( auditivo E processamento E transtornos ) OU TÍTULO-ABS- CHAVE ( distúrbio, E auditivo E processamento ) OU TÍTULO-ABS-TECLA ( OU TÍTULO-ABS-KEY ( processamento E distúrbio , E auditivo ) OU TÍTULO-ABS-KEY ( processamento E distúrbios, E auditivo ) OU TÍTULO - ABS - KEY ( psicoacústico E distúrbios ) OU TÍTULO - ABS -KEY ( transtorno E psicoacústico ) OU TÍTULO-ABS-KEY ( transtorno E psicoacústico ) OU TITLE-ABS-KEY ( psicoacústico E transtorno ) OU TITLE-ABS-KEY ( acústico E perceptivo E transtorno ) OU TITLE-ABS-KEY ( acústico E perceptivo E transtornos ) OU TITLE-ABS-KEY ( transtorno E acústico E perceptivo ) OU TÍTULO-ABS-KEY ( distúrbios, E acústico E perceptivo ( _ _ _ _ _ _ _ _ _ _ _ _ _ _ _ _ _ _ _ _ _ _ _ _ _ _ _ _ _ _ auditivo E compreensão E distúrbio ) OU TÍTULO-ABS-CHAVE ( auditivo E compreensão E ) OU TITLE-ABS-KEY ( compreensão E distúrbio , E auditivo ) OU TÍTULO-ABS-KEY ( compreensão E distúrbios, E auditivo ) OU TÍTULO-ABS-KEY ( transtorno, E auditivo e compreensão ) OU TÍTULO-ABS-KEY ( distúrbios, E auditivo E compreensão ) OU TÍTULO-ABS-KEY ( auditivo E desatenção ) OU TÍTULO-ABS-KEY ( auditivo E desatenção ) OU TÍTULO-ABS-KEY ( desatenção, E auditivo ) ) ) ) E ( ( TÍTULO-ABS-KEY ( surdez ) OU TÍTULO-ABS-KEY ( audição E perda ) , E completo ) OU TITLE-ABS-KEY ( completo E audição E perda ) OU TITLE-ABS-KEY ( audição E perda, E extremo ) OU TITLE-ABS-KEY ( Extremo E audição E perda ) OU TITLE-ABS-KEY ( pré- lingual E surdez ) OU TITLE-ABS-KEY ( surdez E pré- lingual ) OU TITLE-ABS-KEY ( surdez E adquirido ) OR TITLE-ABS-KEY ( adquirido E surdez ) OR TITLE-ABS-KEY ( surdez E permanente ) OR TITLE-ABS-KEY ( permanente E surdez ) OU TITLE-ABS-KEY ( permanentes E surdez ) OR TITLE-ABS-KEY ( audição E perda E permanente ) OU TITLE-ABS-KEY ( permanente , E audição E perda ) OU TÍTULO-ABS-KEY ( surdo E mutismo ) OU TÍTULO-ABS-KEY ( surdo-mutismo ) ) ) OU ( ( TITLE-ABS-KEY ( audição E perda ) OR TITLE-ABS-KEY ( perda E audição ) OU TITLE-ABS-KEY ( hipoacusia ) OU TITLE-ABS-KEY ( hipoacusia ) OU TITLE-ABS-KEY ( audição E deficiência ) OU TITLE-ABS-KEY ( surdez E transitória ) OU TITLE-ABS-KEY ( surdez E transitória ) OU TITLE-ABS-KEY ( transitória E surdez ) OU TITLE-ABS-KEY ( transitória E surdez ) OU TITLE-ABS-KEY ( transitória E audição E perda ) OU TITLE-ABS-KEY ( audição E perda, E transitório ) OU TITLE-ABS-KEY ( perda, E transitório E audição ) OU TÍTULO-ABS-CHAVE ( transitória E auditiva E perdas ) ) ) ) E ( ( TÍTULO - ABS - CHAVE ( idosos ) OU TÍTULO - ABS - CHAVE ( idosos ) ) ) |
| **BVS (04.03.2022)** |
| **N= 1,387** |
| ((Aged ) OR (Elderly)) AND ((Deafness ) OR (Hearing Loss, Complete) OR (Complete Hearing Loss) OR (Hearing Loss, Extreme) OR (Extreme Hearing Loss) OR (Prelingual Deafness) OR (Deafness, Prelingual) OR (Deafness, Acquired) OR (Acquired Deafness) OR (Deafness Permanent) OR (Permanent, Deafness) OR (Permanents, Deafness) OR (Hearing Loss Permanent) OR (Permanent, Hearing Loss) OR (Deaf Mutism) OR (Deaf-Mutism) OR (Hearing Loss ) OR (Loss, Hearing) OR (Hypoacusis) OR (Hypoacuses) OR (Hearing Impairment) OR (Deafness, Transitory) OR (Deafnesses, Transitory) OR (Transitory Deafness) OR (Transitory Deafnesses) OR (Transitory Hearing Loss) AND (Hearing Loss, Transitory) OR (Loss, Transitory Hearing) OR (Transitory Hearing Losses)) AND ((Cognitive Dysfunction ) OR (Cognitive Dysfunctions) OR (Dysfunction, Cognitive) OR (Dysfunctions, Cognitive) OR (Cognitive Impairments) OR (Cognitive Impairment) OR (Impairment, Cognitive) OR (Impairments, Cognitive) OR (Mild Cognitive Impairment) OR (Cognitive Impairment, Mild) OR (Cognitive Impairments, Mild) OR (Impairment, Mild Cognitive) OR (Impairments, Mild Cognitive) OR (Mild Cognitive Impairments) OR (Mild Neurocognitive Disorder) OR (Disorder, Mild Neurocognitive) OR (Disorders, Mild Neurocognitive) OR (Mild Neurocognitive Disorders) OR (Neurocognitive Disorder, Mild) OR (Neurocognitive Disorders, Mild) OR (Cognitive Decline) OR (Cognitive Declines) OR (Decline, Cognitive) OR (Declines, Cognitive) OR (Communication Disorders ) OR (Communication Disorder) OR (Communicative Disorders) OR (Communicative Disorder) OR (Communication Disorders, Developmental) OR (Communication Disorder, Developmental) OR (Developmental Communication Disorder) OR (Developmental Communication Disorders) OR (Childhood Communication Disorders) OR (Childhood Communication Disorder) OR (Communication Disorder, Childhood) OR (Communication Disorders, Childhood) OR (Communicative Dysfunction) OR (Communicative Dysfunctions) OR (Dysfunction, Communicative) OR (Dysfunctions, Communicative) OR (Neurogenic Communication Disorders) OR (Communication Disorder, Neurogenic) OR (Neurogenic Communication Disorder) OR (Communication Disorders, Neurogenic) OR (Communication Disabilities) OR (Communication Disability) OR (Disabilities, Communication) OR (Disability, Communication) OR (Acquired Communication Disorders) OR (Acquired Communication Disorder) OR (Communication Disorder, Acquired) OR (Communication Disorders, Acquired) OR (Auditory Perceptual Disorders ) OR (Auditory Perceptual Disorder) OR (Perceptual Disorder, Auditory) OR (Auditory Processing Disorder) OR (Auditory Processing Disorders) OR (Disorder, Auditory Processing) OR (Disorders, Auditory Processing) OR (Processing Disorder, Auditory) OR (Processing Disorders, Auditory) OR (Psychoacoustical Disorders) OR (Disorder, Psychoacoustical) OR (Disorders, Psychoacoustical) OR (Psychoacoustical Disorder) OR (Acoustic Perceptual Disorder) OR (Acoustic Perceptual Disorders) OR (Disorder, Acoustic Perceptual) OR (Disorders, Acoustic Perceptual) OR (Perceptual Disorder, Acoustic) OR (Perceptual Disorders, Acoustic) OR (Perceptual Disorders, Auditory) OR (Auditory Comprehension Disorder) OR (Auditory Comprehension Disorders) OR (Comprehension Disorder, Auditory) OR (Comprehension Disorders, Auditory) OR (Disorder, Auditory Comprehension) OR (Disorders, Auditory Comprehension) OR (Auditory Inattention) OR (Auditory Inattentions) OR (Inattention, Auditory) OR (Inattentions, Auditory)) |
| **MedRXIV (05.3.2022)** |
| **N= 41** |
| ((Aged ) OR (Elderly)) AND ((Deafness ) OR (Hearing Loss, Complete) OR (Complete Hearing Loss) OR (Hearing Loss, Extreme) OR (Extreme Hearing Loss) OR (Prelingual Deafness) OR (Deafness, Prelingual) OR (Deafness, Acquired) OR (Acquired Deafness) OR (Deafness Permanent) OR (Permanent, Deafness) OR (Permanents, Deafness) OR (Hearing Loss Permanent) OR (Permanent, Hearing Loss) OR (Deaf Mutism) OR (Deaf-Mutism) OR (Hearing Loss ) OR (Loss, Hearing) OR (Hypoacusis) OR (Hypoacuses) OR (Hearing Impairment) OR (Deafness, Transitory) OR (Deafnesses, Transitory) OR (Transitory Deafness) OR (Transitory Deafnesses) OR (Transitory Hearing Loss) AND (Hearing Loss, Transitory) OR (Loss, Transitory Hearing) OR (Transitory Hearing Losses)) AND ((Cognitive Dysfunction ) OR (Cognitive Dysfunctions) OR (Dysfunction, Cognitive) OR (Dysfunctions, Cognitive) OR (Cognitive Impairments) OR (Cognitive Impairment) OR (Impairment, Cognitive) OR (Impairments, Cognitive) OR (Mild Cognitive Impairment) OR (Cognitive Impairment, Mild) OR (Cognitive Impairments, Mild) OR (Impairment, Mild Cognitive) OR (Impairments, Mild Cognitive) OR (Mild Cognitive Impairments) OR (Mild Neurocognitive Disorder) OR (Disorder, Mild Neurocognitive) OR (Disorders, Mild Neurocognitive) OR (Mild Neurocognitive Disorders) OR (Neurocognitive Disorder, Mild) OR (Neurocognitive Disorders, Mild) OR (Cognitive Decline) OR (Cognitive Declines) OR (Decline, Cognitive) OR (Declines, Cognitive) OR (Communication Disorders ) OR (Communication Disorder) OR (Communicative Disorders) OR (Communicative Disorder) OR (Communication Disorders, Developmental) OR (Communication Disorder, Developmental) OR (Developmental Communication Disorder) OR (Developmental Communication Disorders) OR (Childhood Communication Disorders) OR (Childhood Communication Disorder) OR (Communication Disorder, Childhood) OR (Communication Disorders, Childhood) OR (Communicative Dysfunction) OR (Communicative Dysfunctions) OR (Dysfunction, Communicative) OR (Dysfunctions, Communicative) OR (Neurogenic Communication Disorders) OR (Communication Disorder, Neurogenic) OR (Neurogenic Communication Disorder) OR (Communication Disorders, Neurogenic) OR (Communication Disabilities) OR (Communication Disability) OR (Disabilities, Communication) OR (Disability, Communication) OR (Acquired Communication Disorders) OR (Acquired Communication Disorder) OR (Communication Disorder, Acquired) OR (Communication Disorders, Acquired) OR (Auditory Perceptual Disorders ) OR (Auditory Perceptual Disorder) OR (Perceptual Disorder, Auditory) OR (Auditory Processing Disorder) OR (Auditory Processing Disorders) OR (Disorder, Auditory Processing) OR (Disorders, Auditory Processing) OR (Processing Disorder, Auditory) OR (Processing Disorders, Auditory) OR (Psychoacoustical Disorders) OR (Disorder, Psychoacoustical) OR (Disorders, Psychoacoustical) OR (Psychoacoustical Disorder) OR (Acoustic Perceptual Disorder) OR (Acoustic Perceptual Disorders) OR (Disorder, Acoustic Perceptual) OR (Disorders, Acoustic Perceptual) OR (Perceptual Disorder, Acoustic) OR (Perceptual Disorders, Acoustic) OR (Perceptual Disorders, Auditory) OR (Auditory Comprehension Disorder) OR (Auditory Comprehension Disorders) OR (Comprehension Disorder, Auditory) OR (Comprehension Disorders, Auditory) OR (Disorder, Auditory Comprehension) OR (Disorders, Auditory Comprehension) OR (Auditory Inattention) OR (Auditory Inattentions) OR (Inattention, Auditory) OR (Inattentions, Auditory)) |

**Table 1 -** General characteristics of the studies used in the meta-analysis with color/race/ethnicity approach. (N= 3)

| **Characteristic** | **N** | **%** |
| --- | --- | --- |
| **Study design** |  |  |
| Cross-sectional | 3 | 100.0 |
| **Geographic region** |  |  |
| North America | 3 | 100.0 |
| **Diagnosis of hearing loss** |  |  |
| Normal hearing level < 25 dB | 3 | 100.0 |
| **Diagnosis of cognitive decline** |  |  |
| Mini-Mental State Examination | 1 | 33.3 |
| Interview for Cognitive Status in HRS | 1 | 33.3 |
| Consortium to Establish a Registry for Alzheimer’s Disease (CERAD) | 1 | 33.3 |
| **Sample size*** |  |  |
| ≤ 295 | 2 | 66.7 |
| > 295 | 1 | 33.3 |
| **Methodological quality** |  |  |
| High 7 – 9 | 3 | 100.0 |
| **Year of publication** |  |  |
| 2020 | 3 | 100.0 |
| **Funding** |  |  |
| Yes | 3 | 100.0 |

* Median as cut-off point

**Chart 2.** Studies used in the systematic review evaluated the association between hearing loss and cognitive decline in the elderly.

| **Author**  **Year of Publication**  **Country of the study**  **Data source** | **Sudy design**  **Sample** | **Age, Mean (SD) or Range, y** | **Audiometric Assessment** | **Outcome assessment instrument(s)** | **Covariables** | **Methodological quality of the study** |
| --- | --- | --- | --- | --- | --- | --- |
| 01  **ARMSTRONG** et al., 2020  MALAISYA | **Cohort study**  2012 to 2017  **Sample:** 313 | 60-79 years | Poorer hearing was defined by pure-tone average (PTA) of 0.5-4 kHz tones in the better-hearing ear.  ≥25-40 decibels of hearing level (dB HL) in better ear.  ≥41-70 dB HL in better ear.  >70 dB HL in better ear. | Trail-making Test Part B  (TMT-B), Digit Symbol Substitution Test (DSST), California Verbal Learning Test (CVLT) immediate recall, short-delay, and long-delay, Digit Span Forward/Backward, Benton Visual Retention Test, and Mini Mental State Examination (MMSE). | Age, sex, years of educativon, race, and vascular burden. | **8** |
| 02  **BONFIGLIO; UMEGAKI; KUZUYA,** 2020  EUA | **Cohort study**  **Sample:** 172 | 65–95 years |  | Mini-Mental State Examination  (MMSE) |  | **8** |
| 03  **DEAL et al.,** 2021  EUA | **Cross-sectional study**  2016-2017  **Sample:** 3678 | 72-94 years | Better-ear pure tone average of speech-frequency thresholds (0.5-4 kHz) >25 decibels.. | Delayed Word  Recall, Incidental Learning, Logical Memory I, Logical Memory II, Animal Fluency, Boston Naming Test, Word Fluency, Digits Backwards, Digit Symbol  Substitution, Trail Making Test A, Trail Making Test B. | age, education, sex, smoking, diabetes, hyperten sion, and hearing aid use. | **9** |
| 04  **FU et al.,** 2021  EUA | **Cross-sectional study**  **Sample:** 293 | 111 males, M = 70.33 ± 4.90 years; 182 females,  M = 69.02 ± 4.08 years) | The 4FA hearing loss was  classified using the World Health Organization (WHO) grades of hearing impairment (Humes, 2019), respectively, normal hearing—less than 20 dB HL; mild hearing loss—20 to < 35 dB HL; moderate hearing loss—35 to < 50 dB HL; moderately severe hearing loss—50 to < 65 dB HL; severe hearing loss— 65 to < 80 dB HL; profound hearing loss—80 to < 95 dB HL; complete hearing loss—95 dB HL or greater in the better ear. | Hearing Impairment-Montreal Cognitive Assessment  Test (HI-MoCA), and a computerized neuropsychology test battery (CANTAB). | Age, gender, smoking and alcohol consumption, chronic medical history, education years, loneliness and mental health, and PTA thresholds of better ear average across 500 Hz–8 kHz. | **8** |
| 05  **GE et al.,** 2021  EUA | **Case control study**  **Sample:** 295 | 73 years and older | Inabil-  ity to hear sounds of 25 dB at frequencies between 0.5 and 4.0 kHz in either ear. | Five waves of cognitive function data measured by the HRS  version of the Telephone Interview for Cognitive Status in  HRS (2006–2014). | adjusted for survey wave, years of edu-  cation, race, number of health conditions, and physical exercise. | **8** |
| 06  **HAMZA; ZENG,** 2021  EUA | **Cohort study**  **Sample:** 643 | 60–69 years | Defined as a threshold greater than 25 dB HL  based on the unaided better-ear pure-tone average (PTA) of 0.5,  1, 2, and 4 kHz [World Health Organization (WHO), 2008]. | Consortium to Establish a Registry for Alzheimer’s Disease (CERAD)-word learning,  CERAD-animal fluency, CERAD-word list recall, and the digit symbol substitution test  (DSST) in NHANES, and a comparable Hispanic version of these four tests in HCHS. | race, age, sex,  education, pure tone average, hearing aids, and physical well-being | **7** |
| 07  **HARITHASAN et al.,** 2020  Malaysia | **Cross-sectional study**  from July to October 2013.  **Sample:** 229 | 60 years or older | Defined hearing level on the basis of the better  ear 4FA: normal hearing level 4FA ≤25 dBHL, mild hearing loss 4FA 26 to 40 dBHL, moderate hearing loss 4FA 41 to 70 dBHL, or severe  hearing loss 4FA >70 dBHL. | Geriatric Depression  Scale (GDS‐15), Revised University of California at Los Angeles Loneliness Scale (R‐  UCLA), Satisfaction with Life Scale (SWLS), and Mini‐Mental State Examination  (MMSE). | Age, gender, race, marital status, education level, perceived adequacy of income, and health status referred to as nonsensory factors  . | **8** |
| 08  **HARRISON BUSH et al.,** 2015  EUA | **Cross-sectional study**  **Sample:** 894 | Mean age of 73.4  (SD=6.00) years. | Pure-tone air conduction hearing thresholds were measured using a  calibrated GSI-17 (Grason-Stadler Instruments) portable audiometer and TDH-39  (Telephonics Corp.) earphones at 0.5 kHz, 1 kHz, and 2 kHz in each ear. | Mini-Mental State Exam (MMSE; Folstein et al. 1975). | Age, race, gender,  education, diabetes, heart disease and hypertension. | **9** |
| 09  **KIELY et al.,** 2012  EUA | **Cohort study**  **Sample:** 4221 | 50 – 103 years | Pure-tone hearing thresholds were tested for frequencies  between 0.5 and 8 kHz, on up to four occasions over a period of 11 years. | Mini - Mental State  Examination (MMSE) | Sociodemographic and health variables. | **6** |
| 10  **LIM; LOO,** 2018  CHINA | **Cohort study**  August 2016 and January 2017  **Sample:** 111 | Between 55 and 86 years | The average hearing threshold for each ear was calculated based on the 4 pure tone audiometry frequencies (0.5, 1,  2, and 4 kHz). Hearing loss categories were based on the classification used by the American Speech‐Language‐  Hearing Association,24 (normal hearing [NH] ≤25 dB HL, mild 26 to 40 dB HL, moderate 41 to 55 dB HL, moderately‐severe 56 to 70 dB HL, severe 70 to 90 dB HL, and profound >90 dB HL). | Locally (Singapore) adapted English and Mandarin versions of the 30‐point MMSE (adapted by Feng et al.25) and MoCA (adapted by Dong et al.26) | Adjusted for the confounder of age. | **7** |
| 11  **LIN et al.,** 2013  SINGAPORE | **Cohort study**  **Sample:** 2206 | 70 – 79  years. | A pure-tone average (PTA) of hearing thresholds at 0.5 to 4  kHz was calculated for the better-hearing ear. Hearing loss was defined as a PTA exceeding 25 dB per the definition of impairment by the World Health Organization14 (the level at which hearing loss begins to impair daily communication). | Global function) and the Digit Symbol Substitution test (measuring executive function). | Age, sex, race/ ethnicity, and education. | **9** |
| 12  **MAMO et al.,** 2019  EUA | **Cross-sectional study**  2013 to 2019  **Sample:** 250 | Mage = 77 years, age range: 67.3–89.1 years | Hearing thresholds  were measured at octave frequencies between 0.5 and 8 kHz; a speech frequency pure-tone average (PTA) was calculated by taking the average across four octave frequencies: 0.5, 1, 2, and 4 kHz. The PTA from the better hearing ear was  modeled both as a continuous variable and categorized into  Mamo et al.: Speech-in-Noise Performance and Cognition 1007 clinically defined cut-points (normal hearing: ≤ 25 dB HL, mild loss: 26–40 dB HL, moderate/severe loss: > 40 dB HL). | Delayed Word Recall Test, Incidental learning, Logical memory, Word Fluency, Animal Naming Test, Boston Naming Testc, Digit Symbol Substitution Test, Digit Span Backward Test, Trail Making Test Part A e Trail Making Test Part B. | Age, sex, and education, cardiovascular risk factors, as well as depressive  symptoms and premorbid intelligence (WRAT). | **7** |
| 13  **MAMO; WHEELER,** 2021  EUA | **Cohort study**  **Sample:** 144 | Mage = 74 years, age range: 59.8–99.7 | The PTA from the better  hearing ear was used in analyses, and hearing loss categories were defined as no loss (≤ 25 dB HL), mild loss (26–40 dB HL), or moderate/severe loss (> 40 dB HL). | Memory Orientation Screening Test (MOST). |  | **7** |
| 14  **MUDIE et al.,** 2018  EUA | **Cross-sectional stud**  2012 to 2018  **Sample:** 220 | 55–85 years | HI was defined as four-frequency (0.5–4 kHz) pure tone average (PTA) threshold worse than 25 dB in the better ear. | The Geriatric Depression  Scale (GDS), and Mini-Mental State Exam (MMSE; Folstein et al. 1975). | Age, gender, race and number of comorbidities. | **8** |
| 15  **STICKEL et al.,** 2021  CHINA | **Cross-sectional study**  2008 to 2011  **Sample:** 9180 | 45 - 74 years | Hearing impairment of at least mild severity was defined as the pure tone average of 500, 1000, 2000, and 4000 Hz greater than 25 dB hearing level (dB HL) in the better ear. | Brief-Spanish English Verbal Learning Test (episodic learning and memory), and Word Fluency (verbal fluency), and Digit Symbol Subtest (processing speed/executive functioning), and a cognitive composite of the mentioned tests (overall cognition). | Age in years, sex, education Hispanic or  Latino background, anual household income, marital status. | **9** |
| 16  **SUGAWARA et al.,** 2011  CHINA | **Cross-sectional study**  June 2008 and June  2009.  **Sample:** 293 | who at least 50 years old | Used na average threshold over three frequencies (500, 1, 000 and 2, 000 Hz) for the better  ear, defined mild hearing impairment as participants who could not hear below 40 dB (to 25 dB) and moderate to severe hearing impairment as those who could not hear at higher thresholds than 40 dB. | Mini-Mental State Examination (MMSE), the Center for Epidemiologic Studies for Depression (CES-D) scale, Starkstein’s apathy scale (AS) and the Short Form Health Survey Version 2 (SF-36v2). | confounding demographic factors  (age, gender and amount of education). | **8** |
| 17  **WANG et al.,** 2022  CHINA | **Cross-sectional study**  from June to July 2019 - 2021  **Sample:** 1012 | Aged ≥60 years 428 males; average age, 72.61±5.51 years | Defned as more than 40 dB loss  in the better ear. | Mini Mental State Examination (MMSE). | sociodemographic characteristics included sex, age, education level, monthly income, living status, and marital status. | **8** |
| 18  **XU et al.,** 202  **CHINA** | **Cross-sectional study**  from April 2012 to November 2013.  **Sample: 293** | ≥ 60 years  Old | Hearing impairment  was defined as OPTA > 25 dB of the better ear according to the  World Health Organization’s definition of impairment [World  Health Organization (WHO), 1997]. | Measured using the Chinese version of  the Mini-Mental State Examination (MMSE). | sex, age, education, income, smoking, drinking, systolic blood pressure (SBP), total cholesterol (TC), and low-density lipoprotein cholesterol level (LDL-C). | **9** |

**Chart 3.** Assessment of the methodological quality of cross-sectional studies according to NEWCASTLE –OTTAWA: Quality Access Scale, adapted for cross-sectional studies.

| **AUTHOR, YEAR** | **SELECTION** | | | | **COMPARABILITY** | **OUTCOME** | | **TOTAL** |
| --- | --- | --- | --- | --- | --- | --- | --- | --- |
|  | **Representativeness of the sample** | **Sample size** | **Non-respondents** | **Ascertainment of the exposure** | **Analyze Variable control** | **Assesment of the outcome** | **Statistical test** |  |
| MAMO; WHEELER, 2021 | **-** | **-** | ***** | *** *** | ***** | *** *** | ***** | **7/10** |
| HARRISON BUSH et al., 2015 | ***** | ***** | ***** | *** *** | ***** | *** *** | ***** | **9/10** |
| GE et al., 2021 | **-** | ***** | ***** | *** *** | ***** | *** *** | ***** | **8/10** |
| DEAL et al., 2021 | **-** | ***** | ***** | *** *** | *** *** | *** *** | ***** | **9/10** |
| FU et al., 2021 | **-** | **-** | ***** | *** *** | *** *** | *** *** | ***** | **8/10** |
| HARITHASAN et al., 2020 | ***** | **-** | ***** | *** *** | *** *** | *** *** | ***** | **8/10** |
| MUDIE et al., 2018 | **-** | **-** | ***** | *** *** | *** *** | *** *** | ***** | **8/10** |
| STICKEL et al., 2021 | **-** | ***** | ***** | *** *** | *** *** | *** *** | ***** | **9/10** |
| WANG et al., 2022 | **-** | **-** | ***** | *** *** | *** *** | *** *** | ***** | **8/10** |
| SUGAWARA et al., 2011 | **-** | **-** | ***** | *** *** | *** *** | *** *** | ***** | **8/10** |
| XU et al., 2021 | ***** | **-** | ***** | *** *** | *** *** | *** *** | ***** | **9/10** |

**Chart 4.**  Assessment of the methodological quality of cohort studies according to NEWCASTLE –OTTAWA: Quality Access Scale for cohort studies.

| **AUTHOR, YEAR** | **SELECTION** | | | | **COMPARABILITY** | **OUTCOME** | | | **TOTAL** |
| --- | --- | --- | --- | --- | --- | --- | --- | --- | --- |
|  | **Representativeness of the sample** | **Selection of the unexposed cohort** | **Determination of exposure** | **Outcomes does not present at baseline** | **Comparable groups / Control of confounding variables** | **Definition of outcome** | **Sufficient time for the outcome to occur** | **Appropriate follow-up** |  |
| ARMSTRONG et al., 2020 | **-** | ***** | ***** | ***** | *** *** | ***** | ***** | ***** | **8/9** |
| BONFIGLIO; UMEGAKI; KUZUYA, 2020 | **-** | ***** | ***** | ***** | *** *** | ***** | ***** | ***** | **8/9** |
| HAMZA; ZENG, 2021 | **-** | **-** | ***** | ***** | *** *** | ***** | ***** | ***** | **7/9** |
| KIELY et al., 2012 | **-** | **-** | ***** | **-** | *** *** | ***** | ***** | ***** | **6/10** |
| LIM; LOO, 2018 | ***** | ***** | ***** | **-** | *** *** | ***** | **-** | ***** | **7/9** |
| LIN et al., 2013 | ***** | ***** | ***** | ***** | *** *** | ***** | ***** | ***** | **9/9** |
| MAMO et al., 2019 | **-** | **-** | ***** | ***** | *** *** | ***** | ***** | **-** | **7/9** |

**Figure 1 -** Galbraith plot for studies included in the meta-analysis of the association measurement between hearing loss and cognitive decline, regardless of race/color.


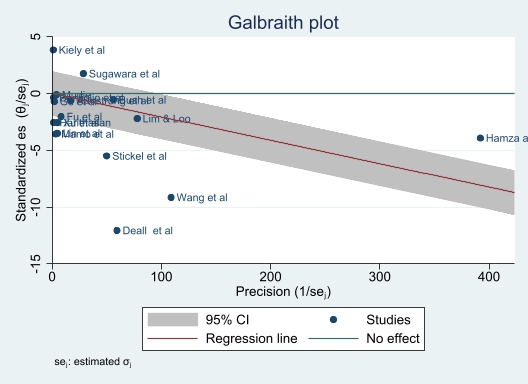


**Figure 2**- Funnel plot of studies exploring the association between hearing loss and cognitive decline, regardless of race/color.


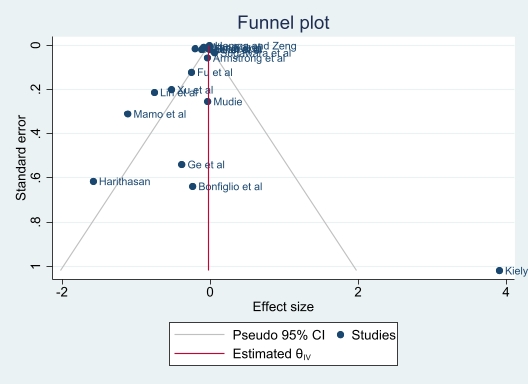

Supplement: S1 File — (DOCX) [file pone.0288099.s002.docx]
